# Revisiting respect for persons: conceptual analysis and implications for clinical practice

**DOI:** 10.1007/s11019-022-10079-y

**Published:** 2022-04-10

**Authors:** Supriya Subramani, Nikola Biller-Andorno

**Affiliations:** Institute of Biomedical Ethics and History of Medicine (IBME), University of Zurich, Winterthurerstr. 30, 8006 Zurich, Switzerland

**Keywords:** Respect, Recognition, Dignity, Autonomy, Clinical practice, Ethics

## Abstract

In everyday conversations, professional codes, policy debates, and academic literature, the concept of respect is referred to frequently. Bioethical arguments in recent decades equate the idea of respect for persons with individuals who are capable of autonomous decision-making, with the focus being explicitly on ‘autonomy,’ ‘capacity,’ or ‘capability.’ In much of bioethics literature, respect for persons is replaced by respect for autonomy. Though the unconditional respect for persons and their autonomy (irrespective of actual decision-making capacity) is established in Kantian bioethics, current argument and debates often revolve around a thin concept of autonomy, focusing on capacity and capability: persons are owed respect because they are ‘rational beings’ or with a focus on ‘agency’ and ‘decision-making abilities.’ However, these aspects alone are insufficient while engaging the concept of respect for persons, particularly in healthcare settings. This paper sets out to explore if the concept of respect for persons—as opposed to a thin concept of autonomy—could help us engage better in healthcare practices. We shall probe the practical value of the experiential aspect of respect—understood as the recognition of persons as respect-worthy through certain dispositions and deliberative acts—by reflecting on instances in clinical practice that tend to be dismissed as negligible or even unavoidable in a stressful environment such as a busy hospital. We shall argue that these instances are far from trivial but carry moral significance and express an unacceptable—disrespectful—attitude that can compromise the moral habitus in hospital settings. In our conclusion, we call for practicing recognition respect in the health professional–patient encounter by focussing on manners, attitudes, and behaviors. Furthermore, we call for continuous medical ethics education programs to address the moral significance of disrespectful behaviors and their manifestations in particular socio-cultural contexts.

## Respect for persons in bioethics

That respect is owed to everyone is a general claim widely acknowledged across disciplines and has been of significance to philosophers across a broad range of moral theories (Downie and Telfer 1969; Cranor 1975; Darwall 1977; Pettit 1989; Buss 1999a; Dillon 2007). To respect a person means recognizing their intrinsic (priceless) worth or dignity, at least from a Kantian perspective (Heubel and Biller-Andorno 2005). In the global crisis caused by the current pandemic, respect has been invoked a lot, e.g., by calling for respect for healthcare workers during the COVID-19 crisis, by stipulating that pandemic response efforts respect human dignity and rights, or by asking to respect a dying person's wish for human company. Nevertheless, understanding respect, meanings, and interpretation may differ among social and cultural contexts.

Patients should be treated as persons, with respect and dignity, which is often emphasized in bioethics and human rights debates (Beach et al. 2007; Dickert 2009; Entwistle and Watt 2013; Henry et al. 2015; Brännmark 2017; Subramani 2018; Árnason 2021). Lysaught (2004) provides helpful intellectual archaeology of 'respect' in bioethics. She suggests that in the early days of contemporary bioethics, respect was focused on persons with and without decision-making capacity. This understanding changed with the Belmont Report (The Belmont Report: Ethical Principles and Guidelines for the Protection of Human Subjects of Research, 1978), which, as Lysaught argues, ascribed respect to de-facto autonomous persons and protection to persons that lack decision-making capacity. Around this same period, the concept of personhood and its interlink to human beings' rights and moral status was highly debated, particularly in the controversies around abortion, assisted reproduction, and brain death (Macklin 1983; Engelhardt 1988). As a result, personhood was criticized as a prescriptive, value-laden term and was more and more avoided. Similarly, bioethics has struggled with the notion of dignity. According to Macklin (2003), the concept of dignity was dispensable, as key bioethical concerns were already covered by respect for persons and respect for autonomy: “the need to obtain voluntary, informed consent; the requirement to protect confidentiality; and the need to avoid discrimination and abusive practices” (Macklin 2003, p. 1419). Other scholars defend respect for human dignity by emphasising the respect for dignity as a universal moral requirement in moral discourse and try to deduct concrete implications for moral action(Árnason 2021; Jacobson 2009; Hofmann 2020). For instance, Jacobson (2007) provides an extensive list of violations of dignity, such as exploitation, dismissal, labelling, bullying, objectification, assault, etc. More recently, few scholars have provided an interpretation of respect for persons by focusing on persons as holders of concrete rights, emphasizing that we can move past a merely theoretical engagement with personhood (Millum and Bromwich 2020).

Unlike many bioethics discourse on human dignity, this paper is not concerned with who (or what form of human life) is considered part of a moral community and why. Instead, it deliberates on what it means to act respectfully and on the practice of respect by healthcare professionals towards patients and family members. Thus, we presume that all (born) human beings are entitled to respect, i.e., patients and their family members deserve respect from their healthcare professionals (and vice versa). We use the term ‘respect for persons’ not to denote who is owed respect and who is not, but as an embodied concept where attitudes, behaviors, and manners within particular contexts as central to our discussion. We contend that an exclusive focus on genuine autonomy as decision-making capacity, which is a thin concept of autonomy (Gutmann 2013; Reis-Dennis 2020), will limit its moral significance and therefore pursue a more expansive notion of autonomy as respect for persons.

Health services research, medical sociology, medical anthropology, and psychological research have established the need for engaging with the concept of respect towards patients, and demand for respectful attitudes and behaviors towards patients from healthcare professionals (Joffe et al. 2003; Lalljee et al. 2007; Bradley et al. 2016; Brown et al. 2018; Lalljee et al. 2007; Clucas and Claire 2010). In bioethics, much of the discussion still focuses on autonomous choice and the decision-making capacity of patients by invoking the ethical principle of respect for autonomy (Donchin 2001; Entwistle et al. 2008, Entwistle et al. 2010; Beauchamp and Childress 2013; Groll 2014; Bullock 2018). In comparison, some scholars critique this notion of respect for autonomy by emphasizing the relational understanding of autonomy within the good clinician–patient relationship and emphasize the capability argument, particularly regarding patients' self-identify and autonomy capabilities (Entwistle et al. 2010). Several scholars have pointed towards the shift in the language of respect in bioethics, where respect for persons is emphasized regarding respect for autonomy and demands acknowledging the broader focus of respect for persons, a recognition of the unconditional value of patients or research participants as persons (Lysaught 2004; Dickert 2009; Henry et al. 2015). Some scholars have engaged respect and dignity under an interrelated framework and have developed a conceptual model for respect for dignity (Henry et al. 2015). Other scholars have presented a set of themes regarding treatment with respect and dignity within the clinical practice as understood by patients and family members, particularly for the intensive care unit (ICU) setting (Aboumatar et al. 2015b; Sugarman 2015; Brown et al. 2018). While these contributions shed light on specific settings, they do not engage much with the theoretical underpinnings of the concept of respect.

In this article, by drawing on Darwall (1977, 2006) and Buss (1999a, 1999b), we engage with the concept of ‘recognition respect’ and its significance to respectful manners, attitudes, and behaviours in clinical practice.

## Recognition respect, respect for persons and appearing respectful: conceptual analysis

Different meanings and accounts of respect have been proposed by various scholars, for instance: a general account on respect as a means of understanding morality itself; Hudson (1980) focus on four types of respect towards the ‘object,’ including obstacle respect, directive respect, institutional respect and evaluative respect; Dillon (1992) who proposed care respect focus on protection and nurturing. Dillon (2007) suggests that although different scholars discuss various kinds of respect, most scholars consider respect as both an attitude and as behaviour. One helpful explanation of the attitude of respect is ‘a relationship between a subject and an object, in which the subject responds to the object from a certain perspective in some appropriate way. Respect necessarily has an object: respect is always for, directed toward, paid to, felt about, shown for some object’ (Dillon 2018, p. 4). In the discussions around the understanding of respect, it is generally thought that respect is owed to the person and demands appropriate behaviour. Applying this to the doctor–patient relationship, both are subjects and objects of respect. In professional medical ethics codes and ethical arguments discussing the doctor–patient relationship, there is a range of concepts such as autonomy, dignity, and rights that demand respect, particularly towards patients.

**Fig. 1:**
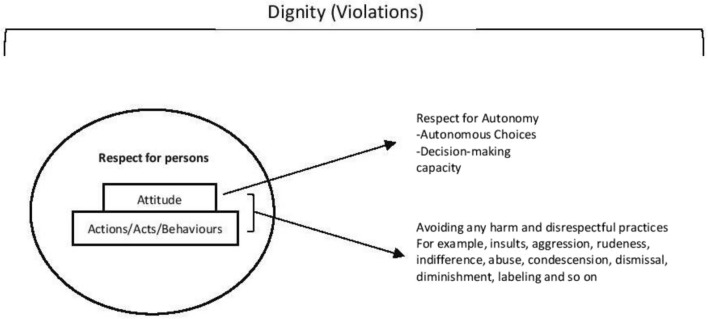
Capturing respect for persons

Often quoted in bioethics literature while discussing respect for persons, Kant's famous account treat ‘persons as ends in themselves’ because they have dignity and are worthy of respect. While discussing respect and critiquing the principle of respect for autonomy, many bioethics works have focused on personhood debates from an end-of-life and dementia lens (Epstein 2013; Nys 2013). Although personhood debates are helpful to particular contexts and studies, they have limitations regarding everyday clinical interactions in hospital settings. Kitwood (1997) and many other scholars (Dewing 2008; Nys 2013) have discussed the limitations of an individualist understanding of personhood and argued for a relational aspect of personhood (Mackenzie and Stoljar 2000). While it may be suggested that respect for persons does not cover all elements and dignity does (Hofmann 2020), we find this needs a critical relook at how the meaning and interpretation of respect for persons is understood. In Hofmann's (2020) review, respect for autonomy and respect for persons are used as one category. However, we suggest distinguishing these two concepts. While dignity is often considered attributional and normative, i.e., as intrinsic to the person, the principle of respect for autonomy emphasizes acknowledging persons as autonomous and focuses on valuing the decision-making capacity of each person. We suggest understanding respect for persons as being responsive to each person's dignity through such attitudes, manners, and behaviors. We shall argue that respect for persons is about how relationships and interactions are governed and regulated through respectful attitudes, manners, and behaviors. Please refer to Fig. 1 to further understand these concepts in this paper.

One influential account, focusing on behaviors while discussing respect, has been articulated by Darwall (1977). We argue here that Darwall's account of recognition respect is beneficial to our context and regarding practicing the principle of respect for persons within the doctor–patient relationship and healthcare settings. While Darwall's work has been widely referred in moral philosophical debates and its application has been present in some psychological and feminist works, it has not been well accounted for in bioethics debates. However, we think it has much to offer to the health care context, particularly when discussing everyday clinical practice, clinical encounters, and doctor–patient relationships. We hope to briefly demonstrate in the following sections the significance of Darwall's work and its interrelation with other scholars who closely engage with the concept of respect for persons.

Darwall (1977) considers two forms of respect: appraisal and recognition. Appraisal respect is positive regard for specific characteristics, skills, or knowledge of objects, which have been determined to have special significance and deserve respect. In his terms, appraisal respect ‘is esteem that is merited or earned by conduct or character’ (p. 122). For example, we may appraise a doctor for her intense efforts for her patients. Recognition respect, on the other hand, is not concerned with the appraisal of individuals, but rather the recognition that we have a moral obligation towards other persons, which we need to acknowledge during the deliberation of our acts (Darwall 1977, 2006). For instance, it is required to respect each individual through attitudes, manners, and behaviors that acknowledge them as persons, whether an older woman in a nursing home or a young woman on the labor ward while giving birth. We can find a similar distinction regarding respect in Kant's writing on ‘Achtung’ (respect). Kant refers to three main kinds of ‘Achtung’: respect for the moral law, the feeling of appraisal respect for a person (reverentia), and the maxim of engaging in recognition-respect, respect in the practical sense (observantia) (Kant 1996).

For our purposes, to adapt respect for persons to healthcare ethics debates during clinical practice, rather than engaging in a detailed analysis of Kant's interpretation of and distinctions between these concepts, we focus on respect as it is expressed in Darwall's account of ‘recognition respect’, which is not unlike Kant's observantia. In the *Metaphysics of Morals*, Kant describes observantia as an action-guiding maxime rather than an emotional response resulting from our comparing our value against that of others. He refers to respect as duty and in this sense as an obligation toward other human beings as they are human beings:

The duty of respect for my neighbor is contained in the maxim not to degrade any other to a mere means to my ends …But in observing a duty of respect I put only myself under obligation; I keep myself within my own bounds so as not to detract anything from the worth that the other, as a human being, is authorized to put upon himself. (Kant 1996, 6:450)

Kant's ‘duty of respect’ refers to the first conjunction of Kant's ‘Formula of Humanity,’ ‘never treat others as means’. He refers to many ways through which one can violate duties of respect for other human beings, such as ridicule, arrogance, and defamation (Kant 1996, 6:465). Darwall's reference to respect is similar to Kant's observantia, where the duty of respect is to be fulfilled by an individual because he or she has dignity and recognizes the need to restrict him- or herself so as not to do so treat others as means. According to Darwall's recognition respect, it is ‘not how something is to be evaluated or appraised, but how our relations to it are to be regulated or governed. Broadly speaking, to respect something in this sense is to give it standing in one's relations’ (Darwall 2006, p. 123). Thus, both conceptions of respect emphasize the practical deliberation of acts and behaviors, and individuals are obliged to perform the duty of respect toward each other. According to Kant and Darwall's understanding of respect, all have a moral obligation to behave and act with recognition respect towards all persons irrespective of evaluating or appraising them as worthy or not, as all persons have intrinsic worth, i.e. dignity. Suppose we adopt this understanding in healthcare interactions and hospital settings. In that case, healthcare professionals are obliged and have a duty to respect patients and family members, not due to evaluative characters or decision-making capacity or by merely focusing on autonomous choices they make during treatment, but because they are persons worthy of respect and possessing dignity.

Recognition respect demands that one act and behave respectfully. We argue, taking forward the work of Subramani (2018; 2020), that one significant way of practicing respect is by avoiding certain acts and behaviors and being aware of micro-inequities—apparently small events or behaviors or actions that are often ephemeral and hard to prove. Because micro-inequities sustains and reproduces the power asymmetry within clinical encounters and raises ethical concern regarding dignity and respect of patients and family members, it creates an institutional ethos that disrespects and marginalizes vulnerable individuals (Subramani 2018). A third person seeing these subtle micro-level disrespectful interactions can see this as outright or overt discrimination who is from different social positioning. Furthermore, the key insight is that in this context the perpetrator behaves differently to different individuals based on the intersectional categories they belong to. For example, physician may behave with affluent and educated patient differently to a patient from lower socio-economic and ethnic group. However, microinequities are hard to object to or prevent or confront by victims who experience and ‘normalize’ it as part of their everyday experience. Elsewhere it has been established that micro-inequities are normalized and legitimized within the hierarchical order of hospital settings (Subramani 2018; 2020a; 2020b). These micro-level acts are not considered overt discrimination or disrespectful behaviors by perpetrators, and the danger of micro-inequities lies in their propensity to be ‘normalized’ or not seen as significant or confront who experience it. While micro-inequities may seem like minor offenses in the lens of most ethical theories, such as deontology or utilitarianism, the “(recognition) respect for persons” lens calls for the *activity of claiming* the dignity of the person. This directs reflections to disrespectful attitudes, acts, and behaviors at micro-level interactions. The concept of “recognition respect” thus urges us to inquire what acts or behaviors are considered (dis)respectful. In the next section, we briefly discuss the significance of a moral attitude and behaviors of respect within healthcare settings.

## Appearing respectful and understanding disrespectful behaviours

As mentioned earlier, much of the work on the conceptual understanding of respect within bioethics debates examines the concepts of respect and dignity together. However, the emphasis is on the sources of the concept of dignity. Also, it is focused on certain measurable and observable behaviors. For instance, Henry et al. (2015) focused on the three sources of a patient's dignity: shared humanity, personal narratives, and autonomy. Brown et al. (2018) focused on a set of behaviors that are perceived as the practice of respect and dignity in ICU care. Beach et al. (2007) attempted to highlight the obligation of medical professionals to fulfill an ‘unconditional duty of respect’ and promote both the cognitive dimension (believing that patients have value) and a behavioral dimension (acting under this belief). They explicitly focus on the genuine attitude of respect.

Many philosophers agree that persons are owed respect and dignity, i.e., she is a ‘person, capable of evaluating her situation for herself and setting her own goals accordingly’ (Buss 1999a, p. 787) but disagrees on what it requires and how it is accomplished (Buss 1999a; Dillon 2007). In most bioethics and healthcare debates, respect is invoked to acknowledge the rights, choices, and care. Most of these studies focussed on the patient's choices, recognizing them as a basic form of respect, and demanded a genuine attitude toward patients. While we agree with this conception of respect, which also includes behaviors and attitudes, the explicit focus on how the patients might feel disrespected is as crucial to the conceptual discussion of respect which is often not of much significance to their conceptual discussion. Buss (1999b) defends the broader conception of the duty to treat people with respect to treat other persons as ends in themselves by emphasizing 'good manners' or treating a person respectfully (p. 795). Thus, we acknowledge the recognition respect concept of experiential beings, which has not been previously engaged in bioethics debates. Furthermore, in this section, by employing the recognition respect concept, we emphasize the role of respectful attitudes, manners, and behaviors through which healthcare professionals show respect to patients and their family members to practice respect in clinical settings.

While philosophers agree on respect, they disagree on what it requires from people, and it becomes clear based on a wide range of health-related research and bioethics studies that the practice and meaning of respect are not straightforward in clinical practice. Studies that highlight disrespect in clinical practice or hospital settings provide us with rich understanding by reflecting on disrespectful behaviors and experiences, for instance, disrespectful treatments such as ‘tailing,’ ‘lack of eye contact,’ and ‘teasing,’ which can also be referred to as micro-inequities, along with macro discriminatory practices within particular contexts (Beagan 2001; Sue et al. 2007; Smith-Oka 2015; Subramani 2018). These disrespectful treatments that disregard and disrespect patients or family members are reproduced, normalized, and sustained in hospital settings. The consequences and overall impact of both micro and macro level disrespectful behaviors on the health of patients, as well as the potential effect on the general wellbeing of those who are receiving end of these treatments, are well established (Clucas and Claire 2010; Cooper 2015; Sokol-Hessner et al. 2015; Subramani 2018).

While being polite or kind is suggested to respect a person, treating people with disrespectful behaviors and manners indicates that they are not perceived as respect-worthy persons. These behaviors and manners are subject to certain social conventions and norms and are part of the culture of particular contexts and settings. Sometimes they are taken for granted or become part of the moral habitus of hospital settings (Subramani 2018; 2020b). However, people are aware of these forms of social order, and members of the context are obliged to comply (Buss 1999b). In line with Buss's understanding of manners of respect, she suggests that, within Kant's framework, people who think that others should be treated with respect will consider themselves obliged to follow the rules by which the group shows its respect for persons. The notion of dignity and the ethical principle of respect for persons is universal (Macklin 1999), but the way respect is shown is based on its context and norms. Thus, we acknowledge that the notion and ethical value of recognition respect are universal, but manners, behaviors, and actions differ in different groups as perceived as appropriate to that context. Ethical universals, therefore, need not be in opposition to cultural values or norms. Instead, ethical universals such as respect are concepts found in all cultures and societies (Macklin 1999, p. 35). This is why ‘appearing respectful’ matters. For instance, if I appear respectful, I manage to align my expression of respect with the cultural context I am acting in so that my intention is perceived. Buss proposes such a relational understanding of appearing respectful:

good manners are essential to acknowledging the intrinsic value of anyone who deserves to be treated with respect. It is precisely because treating people with courtesy is a direct way of acknowledging their dignity that treating them rudely can undermine their belief in their own intrinsic worth. (Buss 1999b, p. 803)

According to Buss, appearing respectful is important and is a way to show respect to persons, in our case, to patients and family members. To consider this within bioethics debates, healthcare professionals may respect patients' right to choose and act ‘autonomously,’ but they can fail to respect patients if they stare at walls, constantly look at the computer, or send text messages during clinical interactions. Thus, it becomes important to understand what set of attitudes, behaviors, and manners healthcare professionals need to avoid and what behaviors and actions patients and family members perceive as disrespectful, rude, offensive, insulting, or impolite within particular settings. We do not here aim to provide any fixed rules or norms but rather emphasize that a moral attitude, behaviors, and manners play significant roles in clinical practice regarding practicing respect and acknowledging the moral duty to communicate or express respect for others. Given the larger structural inequalities and inequities, appearing respectful matters, not just having the attitude of respect but being expressive or communicative through manners and behaviors the respect which is due to the other individual, especially when there is power asymmetry in a relationship. Many studies have discussed and established the treatment considered respectful by patients, such as: being treated as a person, acknowledgment, being treated as family/a friend, being treated as an individual, being treated as important/valuable, and being treated as equal, attentive, patient and family engagement, responsiveness, treating the patient as a person, introductions and greetings, demeanor, and body language (Dickert and Kass 2009; Cooper 2015; Henry et al. 2015; Sugarman 2015). Similarly, some scholars have attempted to assess the treatment of respect (Frosch and Tai-Seale 2014; Aboumatar et al. 2015a; Brown et al. 2018). Although the bioethics and health services literature has thoroughly recognized patients' rights, autonomy, and a patient-centered care approach, persistent asymmetry and inequity remain during clinical encounters in both developed and developing countries (Schnittker and McLeod 2005; Street et al. 2007; Baru et al. 2010; Sen and Iyer 2012; Hall et al. 2015; Goodman et al. 2017; Foster 2019). Many studies of the doctor–patient relationship have established that inequities during clinical interactions occur due to various factors, including patients' language, ethnicity, education, class, gender, location, and caste (Baru et al. 2010; Pilnick and Dingwall 2011; Aronson et al. 2012; Verlinde et al. 2012; Jungari and Chauhan 2017). These studies offer an underlying macro-perspective on issues of inequality and inequity. However, micro-inequities (Anonymous 2018a) within clinical encounters in hospital settings manifest and sustain these inequities and raise ethical questions often overlooked in bioethics debates. Micro-inequities are small and subtle harms, not blatant forms of discrimination within a particular context. These acts, which one cannot pinpoint as outright discrimination, begin a lawsuit, or fight over, are significant to the person receiving such treatment. Generally, they are unfair and unjust actions inflicted on marginalized individuals based on factors such as ethnicity, gender, caste, race, class, location, education, and age (Rowe 1990; Beagan 2001). To illustrate, let us invoke an excerpt which was taken from the study which emphasized the moral significance of micro-inequities in hospital settings: a young woman, when was asked by the researcher about her interaction with the doctor, stated, ‘I keep following them [the doctors] from the ward to their offices and back. They don't even bother to stop for a second and listen to me … They performed surgery again but did not tell me anything about it: why or what. As everywhere else, we are made to keep waiting’ (Subramani 2018). Even yelling and scolding towards patients and family members within certain hospitals and resource-scarce settings becomes subtle and normalized within these social contexts (Subramani 2018). An excerpt from another study on microaggressions captures the disrespectful treatment. The researcher presents her field observation along with her insights to the event and is best captured in this quote ‘The physician addressed her concerns about her fatigue by joking that she would need to get used to a lack of sleep, telling her, “Because you'll never sleep when the baby [comes]. You will get up [all the time to check on it …]. If you are fodonga (slovenly and lazy) you'll just ignore it”. The patient had thought she was 43 weeks pregnant, but the physician's calculation turned out to be 38.5 (40 weeks is the usual length of pregnancy). He turned to her and said, “You see how you lie then!” Then, with a grin, he asked her, “Are you easy?” She blushed and, with a slight stutter that revealed her confusion about the question, answered that she had been with her boyfriend for two and a half years. Continuing to tease her, he replied, “You see, you are not that easy. You did not get pregnant. Maybe you didn't know how”. At the end of this exchange, he winked at me and the female nurse and intern in the room, the audience of his little joke, saying cheekily, “I'm the worst!”’ (Smith-Oka 2015). When we closely read these narratives by considering the social context of the studies, we can identify how the individual experienced disrespect in this micro-interaction. The subtle experiences and the feeling of being ignored and needing to please people in power by ‘tailing them,’ or the joking, winking and teasing by the perpetrators, suggest how patients and family members were humiliated and not considered as respect-worthy through the behaviors and actions of the healthcare professionals. Kant's writings on the duty of respect help us better capture the idea of respect through moral attitude and behaviors:

The respect that I have for others or that another can require from me (observantia aliis praestanda) is therefore recognition of a dignity (dignitas) in other human beings, that is, of worth that has no price, no equivalent for which the object evaluated could be exchanged—Judging something to be worthless is contempt. (Kant 1996, 6:462–463)

Based on many healthcare and bioethics studies on disrespectful behaviors and actions, we argue that healthcare professionals have a moral obligation and duty to respect patients and family members and avoid disrespectful behaviors. Here one can question what disrespectful behaviors are; we refer to any explicit or implicit behaviors such as humiliating, ridiculing, belittling, insulting, etc. (Kant 1996). To elaborate, for instance, we identify humiliating behavior based on how one feels humiliated by that behavior, for instance, tailing, and how this behavior is interpreted as a humiliation by the existing social norms and conventions in a particular social context. In order to understand what set of actions and behaviors they need to practice and avoid, it is crucial that healthcare professionals understand the social context and be self-reflexive in their actions and behaviors to respect patients and family members. Thus, healthcare professionals must adhere to social conventions and norms, including the moral attitude of respectful behaviors, to show respect for patients as persons. Thus it becomes challenging when healthcare professionals work with patients and family members from different cultures or groups with which they are not familiar. This challenge needs further rigorous analysis and research to critically understand how the moral attitude of respect can be practiced in cross-cultural contexts. However, the main emphasis in this paper is that healthcare professionals are ethically obliged to practice recognition respect by avoiding disrespectful behaviors and actions and practicing respect guided by self-reflection on social and moral norms that show moral attitude and act respectfully towards persons, i.e., patients and family members.

## A call for practicing respect: implications for clinical practice

As discussed in earlier sections, respect can be understood in various ways. For instance, disregarding information needs of patients; exposing them to experimental intervention without keeping them informed; making an inappropriate comment on a patient who is unconscious or dead; or in case of overtreatment or overdiagnoses for profit-making, can be seen as disrespecting them as persons in the sense of attitudinal, as well as behavioral if certain behaviors while conveying them signify disrespect. Taking forward the understanding of Downie and Telfer (1969), respect has both cognitive (beliefs, acknowledgments, judgments), affective (ways of experiencing, emotions, feelings), and conative dimensions (dispositions, motivations). Though the central aspect of respect is an attitude, it also has a behavioral component, as suggested in the earlier section where we emphasized appearing respectful. While respect for persons as an ethical principle and value is essential, particularly within doctor–patient relationship, it is just as important for healthcare professionals to demonstrate respect towards patients or family members of patients in clinical practice. In order to demonstrate respect towards patients, many professional guidelines mention ‘respect and dignity’ as part of the code of ethics. While there are variations across countries in their codes on respect, a common theme that they all have is to encourage respecting patients as individuals and respecting their dignity. For instance, Good Medical Practice (2013) by General Medical Council of UK, has a clause, ‘Treat patients as individuals and respect their dignity’, and to ‘Treat patients politely and considerately.’ However, in some countries like India, there is no explicit mention in the Indian Medical Council Professional Conduct, Etiquette and Ethics, Regulations (2002) of respect towards patients. However, there is a guideline regarding conduct during consultation in Clause 4.2 ‘All due respect should be observed towards the physician in charge of the case and no statement or remark be made, which would impair the confidence reposed in him’. Such a professional code of ethics illustrates professional bodies' attempts on rules of manners and conduct. These codes and guidelines are situated within a historical, socio-cultural and political context. Thus appearing respectful and practicing a moral attitude of respect and behaving in respectful ways within a particular society in which they practice becomes essential. Thus it calls our attention to medical education during the training period and part of continuous medical training programs across different stages of a healthcare professional's career.

Some may consider disrespect as minor or subtle communicative issues and unavoidable in stressful clinical settings. However, ample studies suggest that disrespectful treatment or negative experiences in hospital settings jeopardize care for vulnerable patients; thus, disrespect becomes morally significant (Clucas and Claire 2010; Frosch and Tai-Seale 2014; Bradley et al. 2016). Although some scholars have presented a common set of themes of respectful and disrespectful behaviors, which would help us understand how patients perceive these behaviors (Brown et al. 2018), while they help us understand better the disrespectful experiences, we suggest that it is important to also go beyond having a specific list of such rules or homogenous behaviors. We emphasize that being aware of such behaviors within a particular socio-cultural context is important, and one needs to be mindful (Dobie 2007) and reflective and practice accordingly. Certain manners are respectful in some context, whereas it is not in other situations. Hence, the practitioners need to be agile and reflect upon such acts and behaviors. Like many scholars, who have suggested critically looking at the medical education programs, we suggest that these programs consider hidden curricula and facilitate practitioners in reflecting and enhancing their understanding about themselves and their relationships with their patients, thereby achieving respectful culture and reflective patient-centered care. Rather than use and prescribe ethical principles and discuss quandary ethics, the medical ethics education and humanities programs can consider introducing self-reflection and mindfulness about one's attitudes, actions, bias, and language into their curriculum, leading to enhanced care and respect in clinical practice. Being able to communicate with respect towards patients and family members is of utmost significance, and recognition respect is only possible when practitioners practice this through reflecting on their actions and attitudes. For example, while talking to certain ethnic minority groups, practitioners should restrain themselves from making disrespectful remarks. This does not mean that one should oversimplify certain stereotypical views but be sensitive to the socio-cultural background and interact accordingly.

Being aware of certain cultural backgrounds and social histories and reflecting on their attitude and actions would achieve respectful patient care. While recognition respect can be applied in the context of professionals' obligations towards other professionals and colleagues and obligations of patients towards healthcare professionals, in this paper, we have restricted our discussion towards the doctor–patient relationship and achieving patient-centered care by explicitly focusing on the duty of healthcare professionals and their obligation to recognize and practice respect towards patients and their family members in medical practice. While it is easy to acknowledge the significance of respect, it is more demanding to practice respect exactly. Hence, we need to go beyond acknowledgment of the right to autonomous choices by adopting Buss's (1999b) framework to respect by appearing respectful. To illustrate this, let us give an example, a surgeon can give options and accept the patients' decisions, whatever they may be. However, if the surgeon mocks or humiliates the patients for their decisions during the consultative interaction, it shows disrespect on the surgeon's part. While it is essential to discuss autonomous choices, it is just as important to give enough attention to how respect is shown. In other words, as Buss argues, ‘appearing to respect is essential’ (Buss 1999b, p. 805). Thus, it is important to practice respectful attitude, manners, and behaviors to acknowledge and practice' recognition respect. Though medical education programs discuss communication skills and bedside manners, the discussion around respectful manners, sensitivity, and awareness of socio-cultural context needs to be thoroughly acknowledged in medical ethics education programs. This may be achieved, for instance, by self-reflection methods and feedback from patients' groups, etc. The focus on self-reflection methods within medical education programs and using certain methods to be conscious and mindful around these topics requires a large body of work to be done in this field.

## Conclusion

With the help of micro-level interactions, our paper illustrates that it is morally significant to understand (dis)respectful manners, attitudes, and behaviors in healthcare settings. As suggested in the paper, some scholars have emphasized respect for patients by critiquing the mainstream ethical principle of respect for autonomy, focusing on decision-making capacity. In this paper, we attempted to fill the theory–practice gap of respect for persons; we argued for considering recognition respect using Darwall's framework and emphasized the importance of appearing respectful in a particular context to practice respect within healthcare interactions. While further research is needed to probe the complex nature of (dis)respect, particularly in cross-cultural settings, the fact that disrespect, inequality, and injustice stand in the way of effective patient-centered care is undisputed. We believe medical education and continuing education programs should alert students and physicians to the relevance of respect, its conceptual foundations and pitfalls, and its rich phenomenology. We hope our paper will not only prompt further academic debate but can also serve as a source for training the next generation of health care professionals.
